# Compression Properties and Electrical Conductivity of In-Situ 20 vol.% Nano-Sized TiC_x_/Cu Composites with Different Particle Size and Morphology

**DOI:** 10.3390/ma10050499

**Published:** 2017-05-04

**Authors:** Dongdong Zhang, Fang Bai, Liping Sun, Yong Wang, Jinguo Wang

**Affiliations:** 1 Key Laboratory of Automobile Materials of Ministry of Education, Department of Materials Science and Engineering, Jilin University, Changchun 130025, China; ddzhang14@mails.jlu.edu.cn (D.Z.); baifang14@mails.jlu.edu.cn (F.B.); wyong15@mails.jlu.edu.cn (Y.W.); 2 Cosco Logistics (Beijing) Materials Co., Ltd., No. 3 Maizidian West Road, Beijing 100016, China; hljsunlp@163.com

**Keywords:** nano-sized TiC_x_, size and morphology, compression properties, electrical conductivity

## Abstract

The compression properties and electrical conductivity of in-situ 20 vol.% nano-sized TiC_x_/Cu composites fabricated via combustion synthesis and hot press in Cu-Ti-CNTs system at various particles size and morphology were investigated. Cubic-TiC_x_/Cu composite had higher ultimate compression strength (σ_UCS_), yield strength (σ_0.2_), and electric conductivity, compared with those of spherical-TiC_x_/Cu composite. The σ_UCS_, σ_0.2_, and electrical conductivity of cubic-TiC_x_/Cu composite increased by 4.37%, 20.7%, and 17.8% compared with those of spherical-TiC_x_/Cu composite (526 MPa, 183 MPa, and 55.6% International Annealed Copper Standard, IACS). Spherical-TiC_x_/Cu composite with average particle size of ~94 nm exhibited higher ultimate compression strength, yield strength, and electrical conductivity compared with those of spherical-TiC_x_/Cu composite with 46 nm in size. The σ_UCS_, σ_0.2_, and electrical conductivity of spherical-TiC_x_/Cu composite with average size of ~94 nm in size increased by 17.8%, 33.9%, and 62.5% compared with those of spherical-TiC_x_/Cu composite (417 MPa, 121 MPa, and 40.3% IACS) with particle size of 49 nm, respectively. Cubic-shaped TiC_x_ particles with sharp corners and edges led to stress/strain localization, which enhanced the compression strength of the composites. The agglomeration of spherical-TiC_x_ particles with small size led to the compression strength reduction of the composites.

## 1. Introduction

Cu and Cu alloy have been used in electrical conductivities as functional materials because of their unique properties, such as high electrical and thermal conductivities [1,2], as well as good corrosion resistance in the atmosphere and seawater [3,4]. However, their low strength at high temperatures hampers their application. It is well known that TiC_x_ particles possess excellent properties, such as high melting point [5,6], high hardness [7,8], high Young’s modulus [9], low density [10,11], low thermal expansion coefficient [12], and good chemical stability [13,14]. Therefore, adding TiC_x_ particles to Cu matrix makes it possible to possess both high thermal conductivity and electric conductivity in combination with high strength at the same time. Many studies have been conducted on the fabrication of in-situ TiC_x_ particles-reinforced Cu matrix composites [15,16,17,18,19]. For instance, Liang et al. [20,21] studied the in-situ mechanism of TiC_x_ in a Cu-Ti-C system and successfully prepared TiC_x_-reinforced Cu matrix composites. Lu et al. [22] introduced 40–60 vol.% TiC-TiB_2_ with an average particle size of 1.5 μm into molten Cu by in-situ method. They reported that the ultimate compression strength increased by 123% for the composites compared with the unreinforced matrix. Liang et al. [23] investigated the thermal explosion reaction behavior of Cu-Ti-C systems with different Ti and C particle sizes, and the found that the sizes of C particles have a great influence on the ignition temperatures of the system. Wang et al. [24,25] used grounded carbon nanotubes (CNTs), which were treated via high speed ball milling, as carbon source to fabricate nano-sized TiC_x_ particle-reinforced Al matrix composites. Results showed that tensile strength was improved for the ball milling treatment of CNTs. Accordingly, a carbon source with small size is more needed in combustion synthesis.

It is well known that the mechanical properties and electrical conductivity of metal matrix composites are determined by size, morphology, and the distribution of the reinforced particles [26,27,28,29]. Yu et al. [30] proposed that the smaller size of primary Mg_2_Si could improve the ultimate tensile strength of magnesium alloy. Wang et al. [31] fabricated various volume fraction TiC_x_/Al composites with different particle sizes. They found that high volume fraction TiC_x_/Al composites had higher compression strength, while where was no research concerning the effect of TiC_x_ size on the property of in-situ TiC_x_/metal composites. Meijer et al. [32] studied the compression properties of the composites by finite element simulation, and they found that cubic particles could result in stress/strain localization in a unit cell model. However, almost no corresponding experiments were carried out to confirm the simulation results. As reported above, despite the fact that there are a few studies concerning the effect of particles content and sizes on the properties of particles-enhanced composites, the effects of different sizes and morphologies of particles on the properties of in-situ particles-reinforced metal matrix composites are very rare—especially Cu matrix composites.

In this research, 20 vol.% TiC_x_/Cu composites with various particle sizes and morphologies were prepared by combustion synthesis combined with hot press consolidation in a Cu-Ti-CNTs system. Compression properties and electrical conductivity of the composites were investigated. These results provide an important guidance for the application of in-situ nano-sized TiC_x_/Cu composites.

## 2. Experimental Procedure

The used raw materials were commercial powders of Cu (~45 μm), Ti (~25 μm), and CNTs (~10–25 nm in diameter and ~15–100 μm in length). Composites 1, 2, and 3 were fabricated by the method of combustion synthesis and hot press. The composites corresponding to mixing powders are shown in Table 1. The mixed powders in composite 1 consisted of raw CNTs, Ti, and Cu powders. Composite 2 contained ground CNTs, and raw Ti and Cu powders. The composition of powder in composite 3 was complicated, which included the pre-milled ground CNTs and Cu powders as well as raw Ti powder. The stoichiometric ratio of C/Ti was set to be 1:1. The nominal compositions of reactants were 80 vol.% Cu powders. In a cylindrical ceramic inwall container, ground CNTs was first prepared by milling at the speed of 300 r/min for 2 h, and then ground CNTs and Cu powders were pre-milled at the speed of 200 r/min for 8 h, the prepared mixed powders were finally separated and milled at the speed of 50 r/min for 24 h.

After milling, the mixture powders were compressed into cylindrical compacts; the diameter of the compacts was 28 mm, the height was about 40 mm. Then, the cylindrical compacts were placed in a self-made vacuum furnace with hot press with high-purity argon gas (99.999%). When the temperature measured by W5-Re26 thermocouples rose rapidly, the composites were quickly pressed under the pressure of 40 MPa. Then, the vacuum furnace was cooled down via the water-cooling system to room temperature, and the composites were successfully fabricated.

The characterization of phase constitutions in the 20 vol.% TiC_x_/Cu composites were carried out by X-ray diffraction (XRD, Rigaku D/Max 2500PC, Tokyo, Japan) with Cu Ka radiation at the scanning speed of 4°/min. The morphologies of the extracted TiC_x_ particles were examined by field emission scanning electron microscope (FESEM, JSM 6700, Tokyo, Japan) and high resolution transmission electron microscopy (HRTEM, JEM-2100F, Tokyo, Japan). The particle size in the size distribution images was measured one particle by one particle in 10 images by the software of nano-measure. The software gives the data and distribution of particle size. The microstructure of the composites was investigated by scanning electron microscopy (SEM, Evo18, Carl Zeiss, Oberkochen, Germany) with energy dispersive spectrometer (EDS) (Model Link-Isis, Oxford, UK). The mechanical properties were performed on a servo hydraulic materials testing system (MTS, MTS 810, Minneapolis, MN, USA) at a strain rate of 3 × 10^−4^ s^−1^. The compression test was carried out three times every composite, and the middle curve was chosen as the final curve. The electrical conductivities of the TiC_x_/Cu composites were measured through a digital eddy current metal conductivity meter (Sigma 2008b, Xiamen, China) at room temperature, the electrical conductivity results are quoted as % IACS (International Annealed Copper Standard).

## 3. Results and Discussion

**Figure 1:**
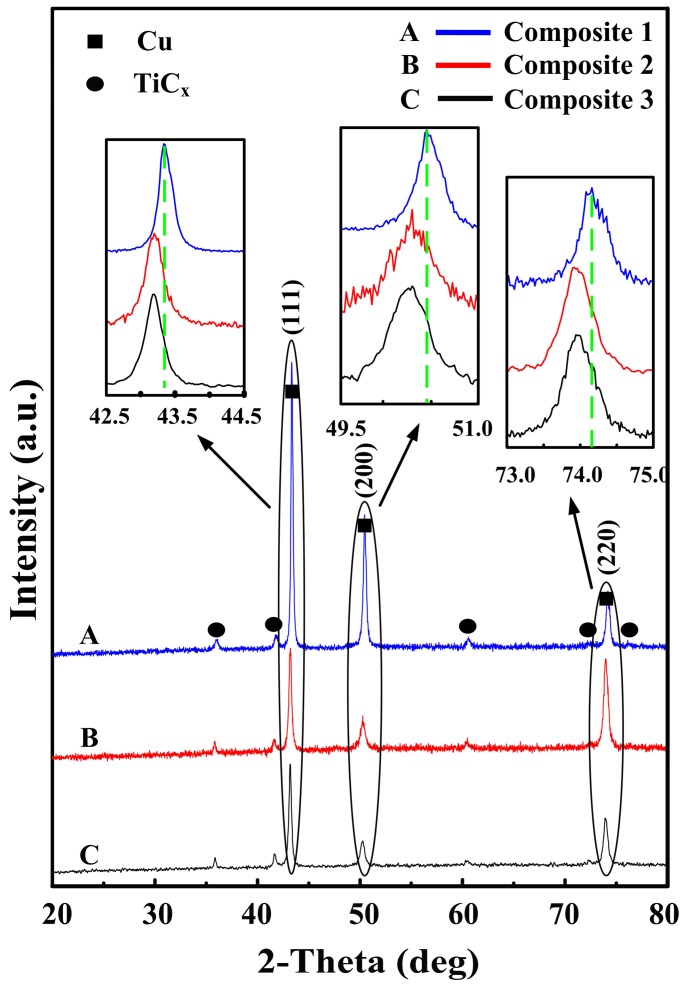
XRD patterns of composites 1, 2, and 3.

**Figure 2:**
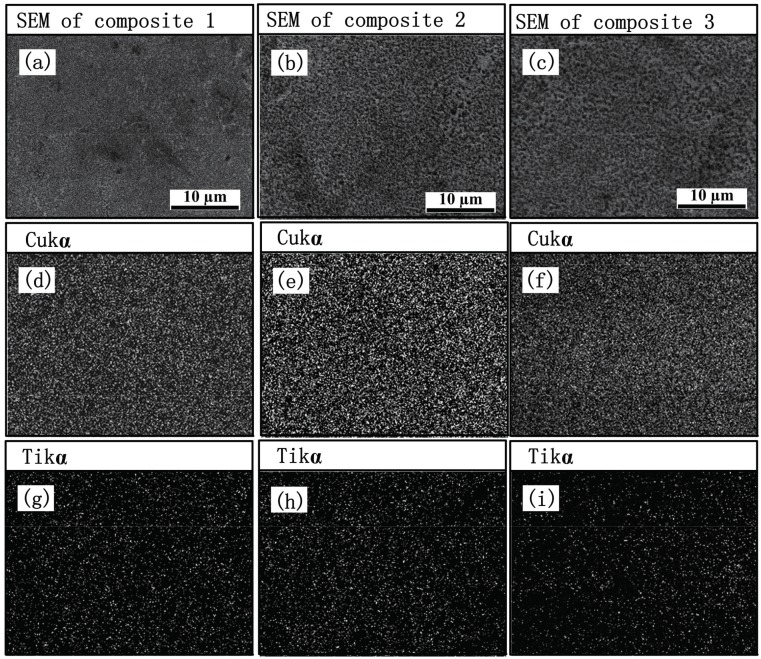
(**a**–**c**) SEM images and (**d**–**i**) energy dispersive spectrometry (EDS) of etched surfaces of composites 1, 2, and 3.

Figure 1 shows the X-ray patterns of composites 1, 2, and 3. It can be found that TiC_x_ and Cu are detected and no obvious intermediate phases are observed, indicating that the 20 vol.% TiC_x_/Cu composites were successfully fabricated via combustion synthesis in combination with hot press. Figure 2 shows SEM images and EDS analysis of etched surfaces of composites 1, 2, and 3. As indicated, the synthesized TiC_x_ particles homogeneously dispersed in the composites (Figure 2a–c). We conjecture that the use of CNTs with high specific surface results in the increase in the contact area between Cu-Ti liquid phase and CNTs, which promotes the in-situ reaction and thus leads to the uniform distribution of TiC_x_ in the composites. EDS analysis of the composites also shows that the various elements exhibit a uniform distribution, as shown in Figure 2d–i.

**Figure 3:**
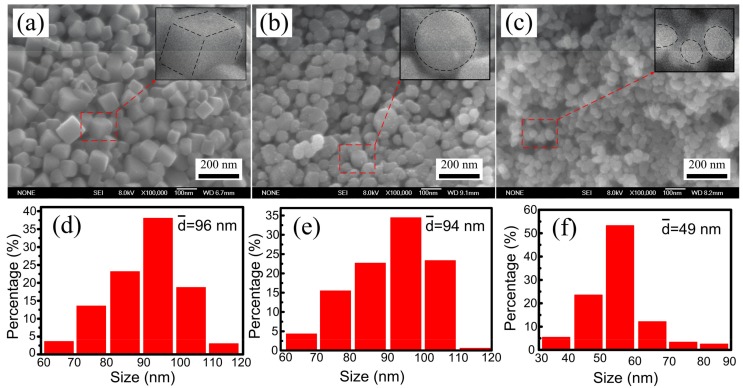
(**a**–**c**) Field emission SEM (FESEM) images and (**d**–**f**) corresponding size distribution of the TiC_x_ particles extracted from composites 1, 2, and 3.

Figure 3a–c shows the FESEM images of the TiC_x_ particles extracted from composites 1, 2, and 3. The size distributions of TiC_x_ particles are shown in Figure 3d–f. As indicated in Figure 3a, most of the TiC_x_ particles in composite 1 are typically cubic in shape, with an average size of 96 nm. The TiC_x_ particles in composite 2 (Figure 3b) and composite 3 (Figure 3c) exhibit spherical and close-to-spherical shape with an average size of 94 nm and 49 nm, respectively. We believed that the decrease of particle size should be attributed to the high-speed pre-milling of Cu and CNTs powders. According to the viewpoint of Liang et al. [20], the mechanism of in-situ reaction is dissolution-precipitation in the Cu-Ti-C system. A small C source is conducive for [C] atoms to dissolve into Cu-Ti liquid phase to form Cu-Ti-C ternary liquid phase, reducing the heat for dissolution of [C] atoms compared with large C source, which would contribute to the decrease of TiC_x_ size.

**Figure 4:**
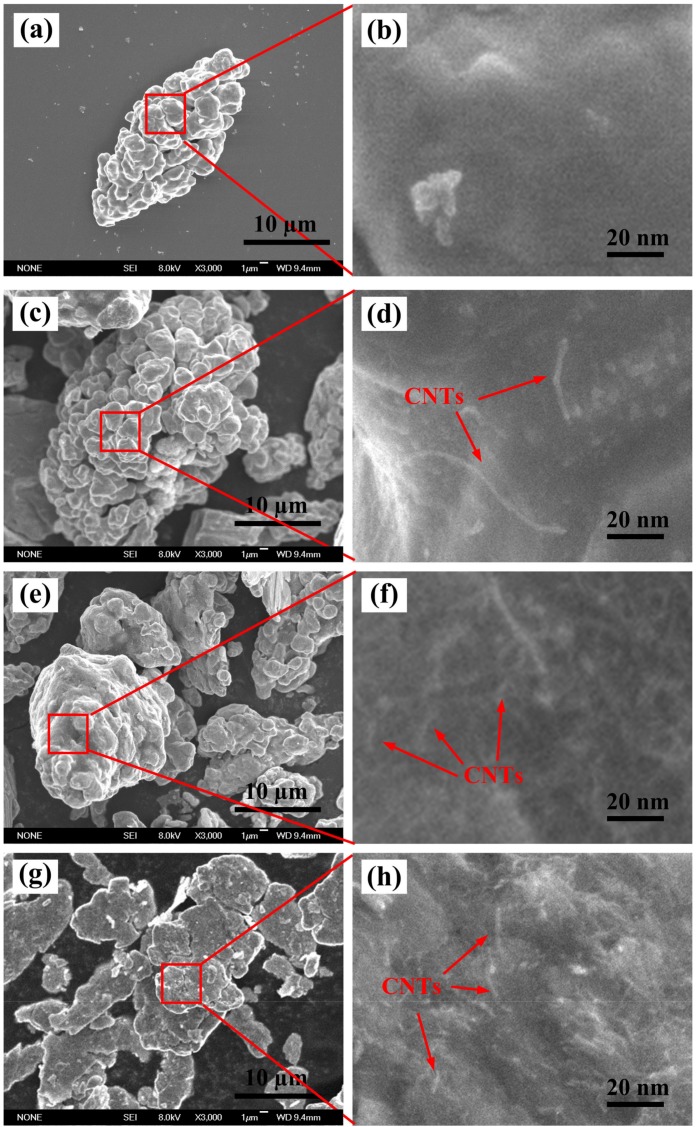
Morphologies of original Cu powders and mixed powders. (**a**) Original Cu powder; (**c**) mixed powders without pre-milling by using original CNTs as C source; (**e**) mixed powders pre-milled by using ground CNTs as C source; (**g**) mixed powders pre-milled by using ground CNTs as C source; (**b**,**d**,**f**,**h**) enlarged images corresponding to (**a**,**c**,**e**,**g**).

Figure 4 shows the FESEM images of original Cu powder (Figure 4a,b) and mixed powders corresponding to composite 1 (Figure 4c,d). It can be seen that the large size distribution of CNTs on the surface of Cu powders is formed. Large CNTs are not conducive to the promotion of the dissolution of [C] atoms into Cu-Ti binary liquid phase, and thus lead to the increase in the concentration of local [C] atoms. Accordingly, the increase in the concentration of local [C] atoms results in high C/Ti mole ratio, which contributes to the formation of cubic-TiC_x_. Figure 4e,f shows the mixed powders corresponding to composite 2. Shortened CNTs uniformly dispersed on the surface of Cu powder can be seen. For the pre-milled ones corresponding to composite 3 (Figure 4g,h), Cu powders were ground into small pieces during the high speed pre-milling process, and shortened CNTs uniformly distributed on the surface of copper powder, which increased the contact area between Cu and CNTs powders, in addition to promoting the in-situ reaction. Finally, the size of TiC_x_ particles decreased.

**Figure 5:**
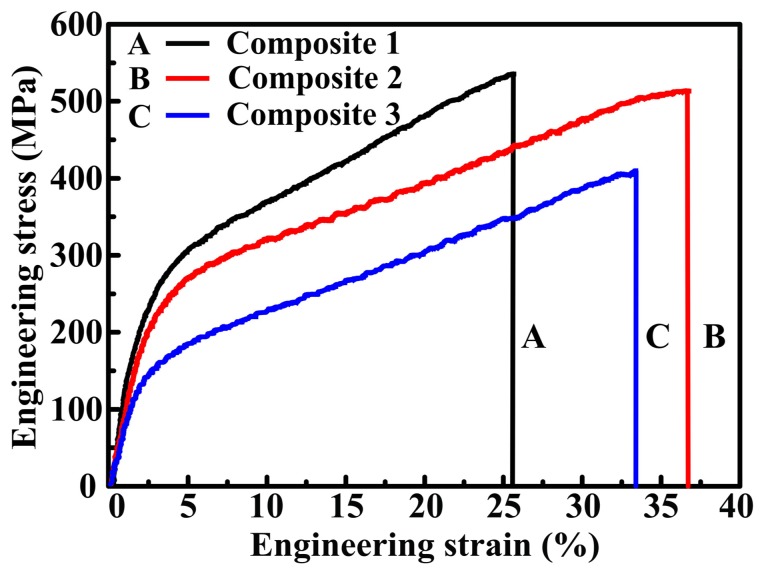
Compression engineering stress–strain curves of composites 1, 2, and 3.

The compression engineering stress–strain curves of composites 1, 2, and 3 are shown in Figure 5. The compression properties data are listed in Table 2. As indicated, the yield strength (σ_0.2_), ultimate compression strength (σ_UCS_), and fracture strain (ε_f_) of 20 vol.% TiC_x_/Cu composites are improved with the increasing particles size. The σ_UCS_, σ_0.2_, and electrical conductivity of spherical-TiC_x_/Cu composite (183 MPa, 526 MPa, and 55.6% IACS) with average size of ~94 nm increased by 17.8%, 33.9%, and 62.5% compared with those of spherical-TiC_x_/Cu composite (417 MPa, 121 MPa, and 40.3% IACS) with particle size of 49 nm, respectively. The σ_0.2_, σ_UCS_, and electrical conductivity of 20 vol.% TiC_x_/Cu composites were improved with the variation of particle shape from sphere to cube. The σ_UCS_, σ_0.2_, and electrical conductivity of cubic-TiC_x_/Cu composite (549 MPa, 183 MPa, and 65.5% IACS) with particle size of 96 nm increased by 4.37%, 20.7%, and 17.8% compared with those of spherical-TiC_x_/Cu composite (526 MPa, 183 MPa, and 55.6% IACS) with particle size of 94 nm. The fracture strains of composites 1, 2, and 3 are 25.9%, 35.7%, and 34.9%, respectively. Cubic-TiC_x_/Cu composite with particle size of 96 nm had the highest σ_0.2_, σ_UCS_, and electrical conductivity among the three composites. Compared with the tensile strength (σ_b_ = 200~250 MPa) and yield strength (σ_0.2_ ≈ 60 MPa) of international annealed copper, the compression strength (σ_UCS_) and yield strength (σ_0.2_) of TiC_x_/Cu composites were improved for the addition of particles with various particle sizes and shapes.

**Figure 6:**
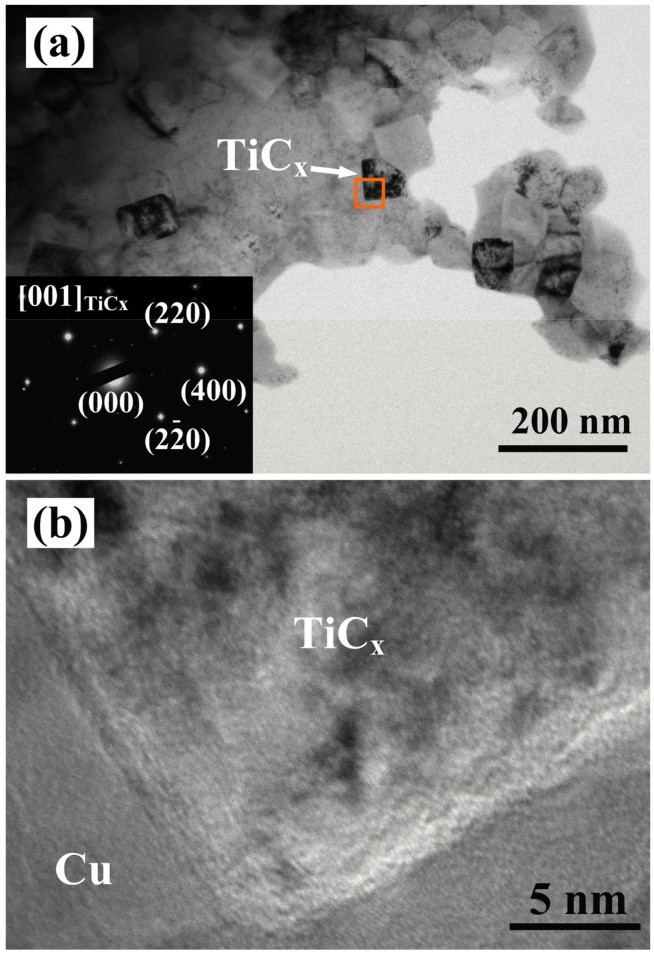
(**a**) TEM images of composite 1 and (**b**) enlarged image of the marked area of composite 1.

Figure 6 shows TEM images of composite 1. The cubic-TiC_x_ particles are surrounded closely by Cu matrix, and there is a good interface between TiC_x_ particles and Cu matrix as shown in Figure 6a,b. The inset images of selected area electron diffraction pattern in Figure 6a corresponding to the [001] zone axis on cubic TiC_x_ particle also show a good interfacial bonding between the TiC_x_ particle and Cu matrix without cracks. According to the viewpoint of Meijer et al. [32], the cubic-shaped reinforced particles with sharp corners and edges lead to stress/strain localization. The elastic metal matrix provided constraint and produced high stress triaxiality for the particles. Accordingly, the initial strain hardening of composites reinforced by cubic-shaped particles is much higher than that of those reinforced by spherical-shaped particles. Following this viewpoint, it can be inferred that the σ_UCS_ and σ_0.2_ of the composites with cubic-TiC_x_ particles can be greatly enhanced by the pinning effect and the stress concentration near edges and corners of cubic-TiC_x_ particles. As for the spherical TiC_x_ particles, TiC_x_ particles with small size have higher surface energy and are easier to agglomerate than TiC_x_ particles with large size [24]. As a result, the agglomeration of TiC_x_ particles played a role as original crack, contributing to the performance reduction of the composite.

**Figure 7:**
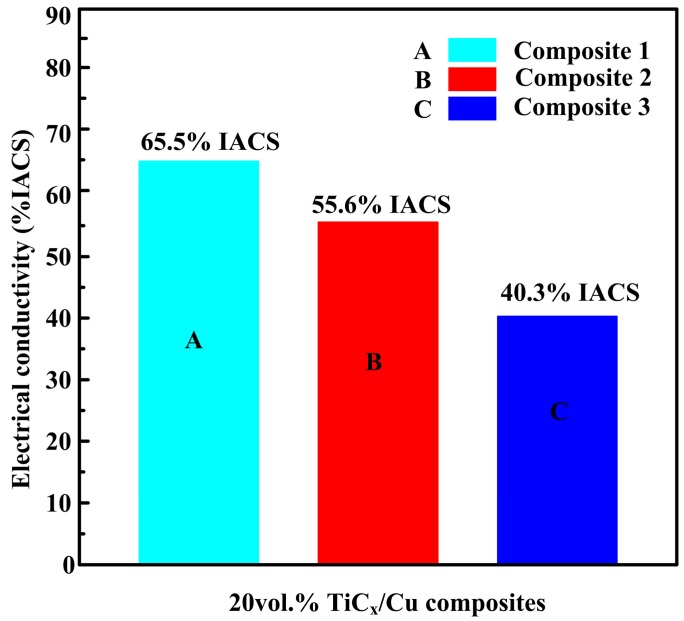
Electrical conductivities of composites 1, 2, and 3.

Figure 7 shows the electrical conductivities of composites 1, 2, and 3. As indicated, the electrical conductivities of the three composites are sensitive to the morphology and size of TiC_x_ particles, which are in the order of composite 1> composite 2 > composite 3. Composite 1, with cubic-TiC_x_ particles, had higher conductivity than that of composite 2, with spherical TiC_x_ particles. The electrical conductivity of composite 2, with large particle size, was higher than that of composite 3, with small particle size.

According to the viewpoint of Zhou [33] et al., the x value of TiC_x_ in substoichiometric analysis corresponding to cubic and spherical TiC_x_ were 0.875 and 0.625, which means that a part of Ti was residual except for the formation of TiC_x_; besides, residual Ti is more in spherical-TiC_x_/Cu composites than that in cubic-TiC_x_/Cu composites on the basis of the substoichiometric analysis. According to Bragg’s law (λ = 2dsinθ), the substitution of Ti for Cu leads to the expansion of the lattice (shift to low angle), since the atomic radius Ti is bigger than that of Cu atoms in Cu lattices, causing the diffraction peaks to shift to a lower angle. The more Ti solution, the lower the peaks will shift to. As described in the inset images of the corresponding area of the X-ray diffraction results in Figure 1, the peaks of Cu in composites 2 and 3 with lower angles mean that residual Ti atoms interact with Cu atoms to form the Cu solid solution; namely, provided more Ti atoms solute in the Cu matrix, the diffraction peak of Cu atoms will shift to lower angles, and the influence of electron scattering will be enhanced. Therefore, we can conjecture that the electrical conductivity of composite 1 (cubic, 96 nm) is higher than those of composite 2 (spherical, 94 nm) and 3 (spherical, 49 nm). As for the spherical-TiC_x_/Cu composite, TiC_x_ particles with smaller size have larger specific surface area, which leads to a larger interface between the TiC_x_ particles and matrix. Therefore, the scattering of the electron for TiC_x_/Cu composites with TiC_x_ particles of 49 nm was thus enhanced, and the electrical conductivity was finally reduced.

## 4. Conclusions

The compression properties and electrical conductivity of in-situ 20 vol.% nano-sized TiC_x_/Cu composites fabricated via combustion synthesis and hot press at different particle size and morphology were investigated. The results show that the morphology and particle size have a pronounced effect on the compression properties and electrical conductivity. The cubic-TiCx composites had the highest σ_UCS_ and σ_0.2_ among the three composites. Cubic-TiC_x_ particles with edges and corners in the composites pinned the movement of the dislocation and grain boundary; moreover, the stress concentration near the edges and corners of cubic-TiC_x_ particles reduced the elongation of composites, both of which led to the improvement of the σ_UCS_ and σ_0.2_ with sacrificing the ε_f_. The σ_UCS_, σ_0.2_, and electrical conductivity of spherical-TiC_x_/Cu composites with particle size of 94 nm were higher than those of spherical-TiC_x_/Cu composites with particle size of 49 nm. The performance reduction of the TiC_x_/Cu composite with decreasing particle size was attributed to the agglomeration of TiC_x_ particles, which played a role as the source of crack, contributing to the reduction of performance. Meanwhile, the increase in the interface between particles and matrix enhanced the scattering of the electron and reduced the electrical conductivity of the composite. The solution of more Ti atoms in Cu matrix for spherical-TiC_x_/Cu composites resulted in the enhancement of electron scattering and the decrease in electrical conductivity compared with the cubic one.

## Figures and Tables

**Table 1:** Components of mixed powders for different composites. CNT: carbon nanotube.

Composites	Carbon Source	Pre-Milled	Mixed Powders
Composite 1	raw CNTs	–	raw CNTs + Cu + Ti
Composite 2	ground CNTs	–	ground CNTs + Cu + Ti
Composite 3	ground CNTs	ground CNTs + Cu	Pre-milled + Ti

**Table 2:** Compression properties of the different composites.

Composites	σ_0.2_/MPa	σ_UCS_/MPa	ɛ_f_/%
Composite 1	$183_{-14}^{+12}$	$549_{-15}^{+12}$	$25.9_{-3}^{+2}$
Composite 2	$132_{-13}^{+9}$	$526_{-13}^{+15}$	$35.7_{-3}^{+5}$
Composite 3	$121_{-14}^{+11}$	$417_{-15}^{+12}$	$34.9_{-2}^{+2}$
